# Impact of orthodontic appliances on patients’ daily lives: a comparison between clear aligners and fixed appliances

**DOI:** 10.1590/2177-6709.30.5.e252562.oar

**Published:** 2026-01-09

**Authors:** Priscilla Carvalhal de OLIVEIRA, Beatriz de Souza VILELLA, Mariana MARTINS E MARTINS, Cláudia Trindade de MATTOS, Vanessa de Couto NASCIMENTO, Oswaldo de Vasconcellos VILELLA

**Affiliations:** 1 Fluminense Federal University, School of Dentistry, Department of Orthodontics (Niterói/RJ, Brazil).; 2 Veiga de Almeida University, School of Dentistry, Department of Orthodontics (Rio de Janeiro/RJ, Brazil).

**Keywords:** Orthodontic appliances. Removable, Orthodontic appliances. Fixed, Quality of life, Aparelhos ortodônticos removíveis, Aparelhos ortodônticos fixos, Qualidade de vida

## Abstract

**Objective:**

This study aimed to compare the impact of treatment with clear aligners versus conventional fixed appliances on the daily lives of orthodontic patients during the first six months and the last six months of treatment.

**Methods:**

The study involved 112 adult patients who were divided into four groups. All participants completed the “Dental Impacts on Daily Living” (DIDL) questionnaire, which included 36 questions and covered five domains including appearance, pain, comfort, general performance and eating/chewing. Descriptive statistics and the Mann-Whitney test were used to analyze the total the scores for each domain, while Fisher’s exact test was used to compare the groups regarding the frequency of “dissatisfied”, “relatively satisfied”, and “satisfied” responses.

**Results:**

The results showed no significant differences (p>0.05) in the total scores or in the scores for each domain, except for the “General performance/Interpersonal relationship” domain during the initial and final phases of treatment with fixed appliances, indicating that patients adapted to the appliance over time. The initial phase of treatment with fixed appliances presented the highest percentage of dissatisfaction.

**Conclusion:**

Both treatments showed a decrease in the level of dissatisfaction reported by patients between the initial and final stages, indicating that adaptation is a crucial factor in the impact of orthodontic appliances on patients’ daily lives. Therefore, orthodontists must be especially attentive to the needs of patients in the period immediately after the device is installed.

## RESUMO

**Objetivo:**

Este estudo teve como objetivo comparar o impacto do tratamento com alinhadores estéticos versus aparelhos fixos convencionais na vida cotidiana de pacientes ortodônticos durante os primeiros seis meses e nos últimos seis meses de tratamento.

**Métodos:**

O estudo envolveu 112 pacientes adultos, divididos em quatro grupos. Todos os participantes responderam ao questionário “Dental Impacts on Daily Living” (DIDL), que incluía 36 perguntas e abrangia cinco domínios, incluindo aparência, dor, conforto, desempenho geral e alimentação/mastigação. Estatísticas descritivas e o teste de Mann-Whitney foram utilizados para analisar a pontuação total de cada domínio, enquanto o teste exato de Fisher foi utilizado para comparar os grupos quanto à frequência de respostas “insatisfeito”, “relativamente satisfeito” e “satisfeito”.

**Resultados:**

Os resultados não mostraram diferenças significativas (p>0,05) nos escores totais ou nos escores de cada domínio, exceto para o domínio “Desempenho geral/Relacionamento interpessoal”, durante as fases inicial e final do tratamento com aparelhos fixos, indicando que os pacientes se adaptaram ao aparelho ao longo do tempo. A fase inicial do tratamento com aparelhos fixos apresentou o maior percentual de insatisfação.

**Conclusão:**

Ambos os tratamentos apresentaram redução no nível de insatisfação relatado pelos pacientes entre os estágios inicial e final, indicando que a adaptação é um fator crucial no impacto dos aparelhos ortodônticos na vida cotidiana dos pacientes. Portanto, os ortodontistas devem estar especialmente atentos às necessidades dos pacientes no período imediatamente posterior à instalação do aparelho.

## INTRODUCTION

Orthodontic treatment can have a significant impact on patients’ daily activities, especially their ability to eat, speak, and smile.^1^ These changes are most noticeable during the first few months^2^ ^,^ ^3^ of treatment, with the greatest impact occurring in the first 24 hours after the device is installed. According to Miller et al.^5^, patients experience a significant decrease in overall quality of life and a substantial increase in pain, which can last up to a week.^5^ ^,^ ^6^ On the other hand, there is a significant improvement in the patient’s quality of life at the end of orthodontic treatment. This improvement, according to Alfawal et al.^7^, occurs regardless of the appliance used.

Many factors can influence the selection of an orthodontic appliance,^5^ but it is important to consider that aesthetics is one of the main factors in the search for treatment.^8^ Conventional fixed appliances are indicated in many cases, especially in childhood and adolescence.^9^ This type of device offers greater control to the orthodontist, as it requires less patient cooperation compared to removable appliances, allowing for more complex mechanics. However, for aesthetic reasons, it is not popular among adult patients.^9^

Recent orthodontic techniques focused on improving aesthetics and facilitating oral hygiene have been developed, with the possibility of removing the appliance when necessary. Clear aligners are one of the available alternatives.^9^ Nevertheless, it is still a more expensive treatment that depends on patient cooperation, often not achieving the same level of finishing as conventional fixed appliances.^10^

Treatments with conventional fixed braces or clear aligners have advantages and disadvantages. On the other hand, the patient’s perception of the treatment is subjective and varies from person to person. They receive information from various sources, and the orthodontist is responsible for managing their expectations and guiding them towards the best treatment option, considering the clinical limitations of each case.^10^

It is important to assess how the patient feels during the adaptation period, as well as at the end of orthodontic treatment, in relation to the daily impact resulting from treatment with these two types of orthodontic treatment. A recent systematic review, of which only two studies were eligible, revealed that the effects of clear aligners and conventional fixed braces on OHRQoL are still inconclusive. The review also highlighted the need for further clinical studies with validated instruments to measure OHRQoL.^11^

Therefore, considering the scarcity of information in literature, this study aimed to gain a better understanding of the patients’ perspective when these two types of treatment are compared. The null hypothesis is that there are no statistically significant differences between fixed devices and clear aligners regarding the impact on patients’ daily lives.

## MATERIAL AND METHODS

This study was approved by the Research Ethics Committee of the Faculty of Medicine of the Fluminense Federal University (number 5.210.254; CAAE 12132919.3.0000.5243). This is an observational cross-sectional study comparing the impact on the daily lives of patients treated with clear aligners and conventional fixed braces. A sample calculation was performed based on the pilot study, which included 20 participants, using the largest standard deviation found (0.33) and a difference of 0.25. The power of the test used was 80% (β=0.2) and α=0.05, which showed that 28 research participants were needed per group: initial fixed appliance (n=28), initial clear aligner (n=28), final fixed appliance (n=28) and final clear aligner (n=28). They were selected according to the treatment phase they were in (initial or final) for each type of appliance (fixed appliance or clear aligner).

**Figure 1:**
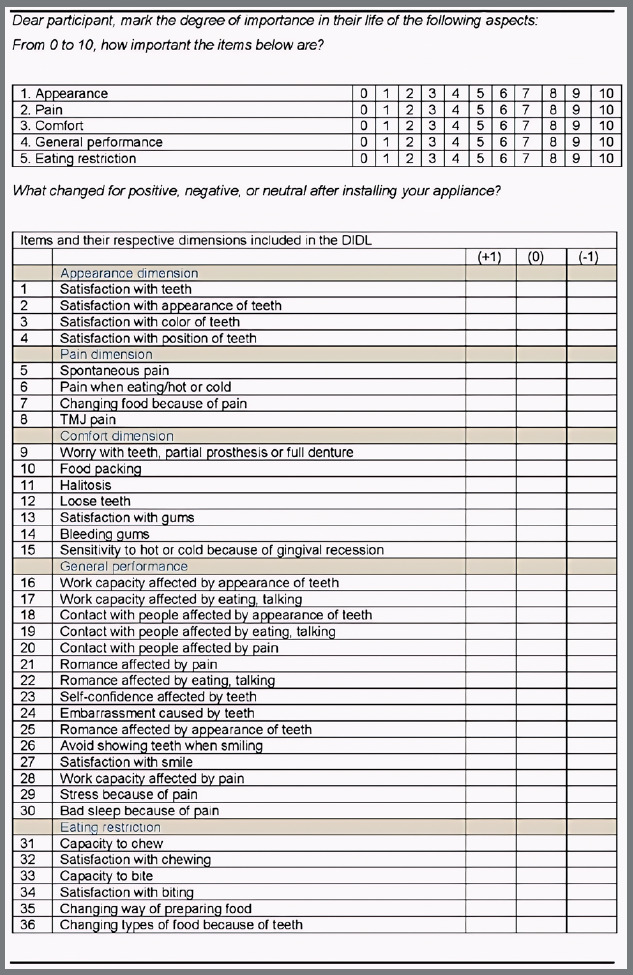
Questionnaire “Dental Impacts on Daily Living” (DIDL).

For this study, the “Dental Impacts on Daily Living” (DIDL) questionnaire (Fig. 1) was used. The questionnaire was previously validated for Brazilian Portuguese by Leao and Sheiham.^12^ The DIDL questionnaire consists of 36 questions and covers five domains that include appearance (individual’s self-image, 4 questions), pain (4 questions), comfort (related to gingival health and absence of food impaction, 7 questions), general performance (ability to perform daily activities normally and interact socially, 15 questions) and eating/chewing (6 questions).

During the research, each participant was asked to rate the importance they placed on different domains on a scale of 0 to 10. This rating helped to assign the relative importance of each domain, resulting in an adjusted score that was more specific to each participant. In each domain, the participants reported how the use of orthodontic appliances affected their daily lives. A score of zero (0) was assigned when there was no interference, a positive score (+1) when there was an improvement, and a negative score (-1) when there was a worsening in the questionnaire question.

The score for each domain was obtained by adding up the impacts attributed by participants to each question and dividing them by the number of questions in each category.

To establish the total score, the domain scores were added and a specific weight was assigned to each: [(sum of appearance question scores/number of appearance questions) x weight assigned to appearance] + [(sum of pain question scores/number of pain questions) x weight assigned to pain] + [(sum of comfort question scores / number of comfort questions) x weight assigned to comfort] + [(sum of performance question scores / number of performance questions) x weight assigned to performance] + [(sum of eating question scores / number of eating questions) x weight assigned to eating] = total score, according to the formula below:

$$\begin{array}{c} \left[\left(\frac{\sum \;s.\;apear\;}{\sum n^{\circ }\;apear\;}\right)\;xweig\;\right]+\left[\left(\frac{\sum \;es.\;pain\;}{\sum n^{\circ }\;pain\;}\right)\;xweig\;\right]+\left[\left(\frac{\sum \;es.\;comf\;}{\sum n^{\circ }\;comf\;}\right)\;xweig\;\right]\\ +\left[\left(\frac{\sum \;es.\;perf\;}{\sum n^{\circ }\;perf\;}\right)\;xweig\;\right]+\left[\left(\frac{\sum \;es.eat\;}{\sum n^{\circ }\;eat\;}\right)\;xweig\;\right]=\;TotalScore\; \end{array}$$

Each domain was assigned a value from 0 to 10 by the participant, representing its importance. To calculate each domain’s specific weight, this value was divided by the sum of all the values assigned to all domains. This ensured that the total sum of each respondent`s weights was always equal to 1. Individual scores ranged from -1 to 1, with scores below 0 indicating dissatisfaction, scores between 0 and 0.7 indicating relative satisfaction, and scores above 0.7 indicating satisfaction. Scores below 0 indicated that more than half of the impacts were negative.

The questionnaires were completed by participants with prior guidance from the researchers, without identifying their names. Recruitment was conducted through the evaluation of procedure forms, which identified eligible participants to be invited to participate in the study. 

Interviews to complete the questionnaires were conducted in a private location, ensuring participant privacy. The average completion time was 15 minutes. Participants were recruited from the Clinic of the Graduate Course in Orthodontics at Fluminense Federal University.

As inclusion criteria, participants of both sexes had to be over 18 years old, treated exclusively with fixed metal braces or clear aligners and be in the period between the first and last six months of treatment.

Participants with cognitive disorders, craniofacial anomalies, active caries or those using auxiliary devices (such as headgear) were excluded.

## STATISTICAL TREATMENT

Differences between groups related to age and sex distribution were tested using ANOVA and chi-square tests, respectively. 

Descriptive statistics, including median and interquartile range, were used to analyze the total scores and each domain for each device and treatment phase. The Mann-Whitney test was used to compare scores between groups and time points.

The frequencies of dissatisfaction, partial satisfaction, and complete satisfaction were calculated for each device and time point, and their differences were compared using Fisher’s exact test. Survey graphs were generated.

## RESULTS

One hundred and twelve participants responded to the questionnaires (28 in each group). Of these, 76 (67.8%) were women and 36 (32.2%) were men. The average age was 36.5 years, ranging from 18 to 68 years.

In the fixed appliances group, 39 were women (69.6%) and 17 were men (30.04%), aged between 18 and 68 years. In the aesthetic aligners group, 37 were women (66%) and 19 were men (34%), aged between 19 and 65.

Table 1 presents the demographic characteristics for each group and the absence of statistical differences between the groups related to age or sex distribution.

**Table 1:** Demographic characteristics for each group and statistical differences between groups related to age or sex distribution.

	Group 1	Group 2	Group 3	Group 4	p*
Age - mean (SD)	33.4 (13.51)	38.8 (11.48)	34.4 (11.64)	39.5 (9.00)	0.118
Sex - n (%)					
male	9 (32%)	10 (35.5%)	8 (28.5%)	9 (32%)	0.955
female	19 (68%)	18 (64.5%)	20 (71.5%)	19 (68%)	

*p-value of differences between groups: age difference according to ANOVA test; sex distribution difference according to chi-square test.

## ASSESSMENT OF TOTAL SCORES AND SCORES FOR EACH DOMAIN

We accepted the null hypothesis, except for the “General performance” domain.

When comparing the initial and final values of each group there was a statistically significant difference (0.025) only for the “general performance” domain, referring to the fixed appliance, in the initial and final phases of treatment (Table 2).

**Table 2:** Medians, interquartile ranges, and p-values for the types of appliances used for each domain studied, at the two treatment times (initial and final ).

		ALIGNER	FIXED AP.
		Median (IQR)	Median (IQR)
Appearance	Initial	0.15 (0.06)^a^	0.12 (0.19)^a^
	Final	0.16 (0.04)^a^	0.17 (0.06)^a^
	p	0.687	0.080
Pain	Initial	0.00 (0.00)^a^	0.00 (0.04)^a^
	Final	0.00 (0.00)^a^	0.00 (0.00)^a^
	p	0.774	0.470
Comfort	Initial	0.03 (0.06)^a^	0.31 (0.07)^a^
	Final	0.03 (0.05)^a^	0.05 (0.11)^a^
	p	0.771	0.764
Performance	Initial	0.03 (0.07)^a^	0.01 (0.06)^a^
	Final	0.04 (0.06)^a^	0.04 (0.08)^a^
	p	0.287	0.025*
Eating	Initial	0.00 (0.09)^a^	0.00 (0.23)^a^
	Final	0.00 (0.06)^a^	0.00 (0.12)^a^
	p	0.714	0.194
Total	Initial	0.27 (0.30)^a^	0.16 (0.53)^a^
	Final	0.24 (0.13)^a^	0.32 (0.30)^a^
	p	0.801	0.105

Equal letters on the same line indicate a statistically non-significant difference (p < 0.05).

## SATISFACTION INDEX ASSESSMENT

The distribution of the degree of satisfaction, categorized as dissatisfied (-1 to 0), partially satisfied (0 to 0.7), and satisfied (0.7 to 1), was analyzed in Table 3 for all treatment categories (initial fixed appliance, initial aligner, final fixed appliance, and final aligner). Fisher’s exact test showed a statistically significant difference (p=0.001) in the distribution of satisfaction levels.

**Table 3:** Distribution of all the categories based on the degree of satisfaction, as per the Fisher exact test (p=0.001). The abbreviation IFA stands for initial fixed appliance, ICA for initial clear aligner, FFA for final fixed appliance, FCA for final clear aligner, DISSAT for dissatisfied, PART SAT for partially satisfied, and SAT for satisfied.

SATISFACTION		IFA	ICA	FFA	FCA	TOTAL
DISSAT.	n	10	4	3	0	17
(-1,0 - 0,0)	(%)	(35.7)	(14.3)	(10.7)	0	(15.2)
PART. SAT.	n	18	23	23	28	92
(0,0 - 0,7)	(%)	(64.3)	(82.1)	(82.1)	(100)	(82.1)
SAT.	n	0	1	2	0	3
(0,7 - 1,0)	(%)	0	(3.6)	(7.1)	0	(2.7)
TOTAL	n	28	28	28	28	112
	(%)	(100)	(100)	(100)	(100)	(100)

**Figure 2:**
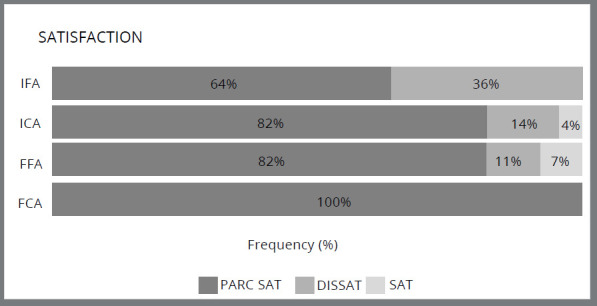
Difference in the percentage distribution of satisfaction in each category (p=0.001).

Figure 2 shows the frequency distribution in terms of satisfaction level. Participants in the initial phase of treatment with fixed braces had the highest percentage of dissatisfaction (38%). No participant reported satisfaction in this group, as well as in the group in the final phase of treatment with aligners.

The initial aligner and final fixed appliance groups were the only categories with a percentage of satisfied participants (4% and 7%, respectively). In the final aligner category all participants were partially satisfied.

## DISCUSSION

Knowledge about patients’ perceptions of conventional fixed appliances and clear aligners will allow orthodontists to have a more realistic view of their likely experiences during orthodontic treatment.

Orthodontic problems can involve aspects of facial aesthetics and masticatory function. According to Bernabé et al.,^13^ nearly a quarter of Brazilian adolescents reported difficulties with eating, speaking, and smiling due to the use of orthodontic appliances in their daily lives.

The DIDL Index questionnaire was used in this study because it assigns weights to the different “domains” assessed, reflecting their respective importance in the individual’s life. The questionnaire was validated for Brazilian Portuguese by Leão and Sheiham,^12^ making the results more specific to each participant.^14^ Furthermore, the DIDL Index accurately captures the impact of these aspects on patients’ daily lives, as Maia et al.^15^ pointed out.

There was no statistically significant difference when evaluating the total scores and the scores for each domain, in the initial and final phases, in the treatments with clear aligners and fixed appliances. The exception was the “General performance” domain which compared treatment with fixed appliances between the initial and final phases. This domain included questions about personal and professional relationships affected by speech, pain, difficulty eating, and satisfaction with the smile.

This result may be related to adaptation to the device over time. At the end of treatment, patients are more satisfied with their smiles and speech due to the correction of the malocclusion. They are even more accustomed to the appearance of the device. A similar result was observed by Chen et al.,^1^ who found that patients’ OHRQoL worsened during treatment compared to the pre-treatment phase (up to one month after the device installation). As they reported, the treatment resulted in a significant improvement in Oral Health-Related Quality of Life (OHRQoL). 

When analyzing the degree of patient satisfaction (as shown in Table 3 and Fig. 2), it was found that the group treated with “initial fixed appliances” had the highest percentage of dissatisfied patients. On the other hand, the group “final clear aligners” was the only one that did not have any dissatisfied patient. A study conducted by Alfawal et al.^7^ also reported higher satisfaction levels among patients treated with clear aligners as compared to those treated with fixed appliances, both during the initial six months of treatment and after treatment. Similarly, Sharma et al.^16^ found that although there was no significant difference in satisfaction levels between adolescents undergoing treatment with fixed appliances or clear aligners, the adaptation time was longer for the former group, when analyzing the entire active treatment period.

According to Flores-Mir et al.^17^ study, both patients treated with orthodontic braces and those treated with clear aligners reported high levels of satisfaction immediately after braces removal. These findings are particularly significant given that the authors used the same Dental Impact on Daily Living (DIDL) questionnaire as in the present study. This is possible because patients were satisfied with the completion of the treatment. However, the present study evaluated patients only while they were using the device, which explains the difference in results.

In the “Appearance” domain, in the questions “Your satisfaction with your teeth” (in general) and “Your satisfaction with the position of your teeth”, the “initial fixed appliance” group was the only one to show negative scores, and the one that presented the lowest number of positive scores. This result suggests that these patients were more dissatisfied with their appearance after orthodontic appliances were bonded than the others. Other authors^7^ ^,^ ^18^ have concluded that patients treated with clear aligners were more comfortable and satisfied with their appearance than those who used fixed appliances, which corroborates the findings of the present study.

In the “Comfort” domain, the 28 patients in the clear aligner group reported no breathing interference in the final phase of treatment, compared with 16 in the fixed appliance group. Eight patients in the fixed appliance group reported gingival bleeding in the final phase, compared with only two in the aligner group. Jiang et al.^19^ and Wu et al.,^20^ in systematic reviews, also found that clear aligners were better for periodontal health when compared to fixed appliances. Similarly, Karkanechi et al^21^ concluded that clear aligners facilitate oral hygiene, suggesting that this type of treatment should be indicated for adult patients at risk of periodontitis. Other studies^22^ ^,^ ^23^ have found that orthodontic therapy with aligners is associated with improved short-term oral hygiene. Wang et al.^24^ reported that, in general, orthodontic treatments, both with fixed appliances and clear aligners, caused dysbiosis of the oral microbiome. However, in terms of microbiome composition and functional aspects, aligners did not outperform fixed appliances. Therefore, the better performance of aligners in terms of periodontal health may not be due to oral microbial conditions, but rather to the ease of cleaning.

The “General performance” domain includes questions such as “Showing teeth when smiling” and “Your satisfaction with your smile”. Both in the initial and final phases of treatment, fixed appliance presented the highest number of negative scores. This implies that patients don`t feel very confident smiling with braces installed. In a study by Sharma et al.,^16^ it was found that although there was no difference in the mean, there was a difference in the distribution of responses regarding “feeling confident”. Almost half of the aligner group reported feeling confident all the time, compared with only 9.4% of the braces group.

In general, the questions relating to speech showed that the patients treated with fixed braces were more dissatisfied than the group treated with clear aligners at the beginning of the treatment. However, at the end of treatment, there was a reversal; the group treated with fixed braces showed a higher level of satisfaction, probably due to the greater adaptation of these patients to the fixed braces. Fraundorf et al.^25^ observed that patients treated with clear aligners had significant speech impairment, which did not return to normal after two months of treatment, despite adaptation. Melo et al.^26^ also found changes identified by a speech-language pathologist at the beginning of treatment, but only in the group treated with aligners. Orthodontists should inform patients that speech alterations may occur temporarily during treatment with clear aligners.

After analyzing the responses related to eating habits, it was found that the group undergoing treatment with fixed braces in the initial phase had the highest number of dissatisfied patients. However, in the final phase, the same group reported a higher level of satisfaction.

On the other hand, the aligner-treated groups had a higher number of neutral responses in both phases, indicating no significant difference in eating or chewing ability after installation. These patients likely didn’t experience any difference after the aligners were installed because they had to remove them during meals. This result is consistent with the observations of Alfawal et al.,^7^ who noted that patients who received clear aligners reported greater comfort during chewing, a more satisfying diet, and fewer interruptions during meals than those who received fixed appliances. Similarly, Alajmi et al.^18^ found that patients treated with conventional fixed appliances reported restrictions in the amount and types of foods they felt comfortable eating, as well as greater limitations in chewing, compared to patients treated with clear aligners. Sharma et al.,^16^ although they found no significant difference, observed that the group treated with fixed appliances was 2.7 times more likely to report difficulty in eating foods they liked, while most patients treated with aligners reported never having had any difficulty. Furthermore, 8.1% of the fixed appliance group reported having trouble eating all the time, compared with 0% of the aligner group. These results were confirmed by Duarte et al.,^27^ who concluded that the activation/installation of the fixed appliances temporarily reduces masticatory performance and bite force.

In the “Pain” domain, both groups presented similar responses. In a randomized clinical study conducted by Casteluci et al.,^28^ it was observed that the type of device did not affect pain intensity during any period evaluated, from installation to six months later. In both groups, pain levels gradually decreased after the second month of treatment. However, Alfawal et al.,^7^ in a similar study, concluded that patients treated with clear aligners experienced less pain than the fixed appliance group during the first week of treatment. Alajmi et al.^18^ observed that most patients in both groups experienced pain for a few days after activation. Patients treated with aligners felt more pressure, while those treated with fixed braces reported throbbing and dull pain. The average pain level was almost nearly the same in both groups, but patients treated with fixed braces reported higher painkiller consumption. Cardoso et al.,^29^ in a systematic review, concluded that patients treated with clear aligners appeared to experience lower pain levels than those treated with fixed appliances during the first few days of treatment. No significant changes were observed after a three-month period.

The results of these studies may have been affected by the subjective nature of pain. Furthermore, it’s worth considering the effects of adaptation to the perceived “tightness” sensation after the activation sessions, as well as the fact that it’s no longer a surprising sensation as it was at the beginning of the treatment.

While providing valuable insights, this study is limited by its cross-sectional design. A longitudinal study would present a more reliable set of data. Therefore, it is recommended that further research be conducted by comparing the initial and final stages of the two types of orthodontic treatments. Additionally, the difficulty in comparing our results with data from studies that used different questionnaires was one of the limitations of the present research.

Despite limitations, this study is the first in which participants were treated exclusively with fixed metal braces or clear aligners, and data were collected between the first and last six months of treatment.

## CONCLUSIONS

Although patients treated with fixed appliances initially reported a higher percentage of dissatisfaction, both groups showed a reduction in dissatisfaction in the final stages of the treatments. Therefore, adaptation is a crucial factor in the impact of orthodontic appliances on patients’ daily lives.
